# Recreational Screen Time Use among a Small Sample of Canadians during the First Six Months of the COVID-19 Pandemic

**DOI:** 10.3390/ijerph182312664

**Published:** 2021-12-01

**Authors:** Paige Coyne, Zach Staffell, Sarah J. Woodruff

**Affiliations:** Department of Kinesiology, Faculty of Human Kinetics, University of Windsor, Windsor, ON N9B 3P4, Canada; staffel@uwindsor.ca (Z.S.); woodruff@uwindsor.ca (S.J.W.)

**Keywords:** COVID-19 pandemic, recreational screen time, longitudinal, sedentary behaviour

## Abstract

(1) Background: The coronavirus (COVID-19) pandemic has caused disruptions in the daily lives of individuals in Canada. Purpose: Examine how total and specific (i.e., watching television, using social media, going on the Internet, playing video games, and engaging in virtual social connection) recreational screen time behaviours changed throughout the first six months of the COVID-19 pandemic, in comparison to pre-pandemic levels; (2) Methods: Sixty four Canadians (mostly Caucasian, female, age range = 21–77 years) completed monthly surveys from April to September of 2020; (3) Results: A one-way repeated measures analysis of variance (RM-ANOVA) and subsequent post hoc analysis revealed that total recreational screen time was statistically higher in late March/April (292.5 min/day ± 143.0) and into May, compared to pre-COVID-19 (187.8 min/day ± 118.3), before declining in subsequent months; (4) Conclusions: Generally, specific recreational screen time behaviours, such as time spent watching television, followed the same trend. Future studies with larger sample sizes and from other countries examining recreational screen time behaviours longitudinally over the pandemic are still needed to allow for greater generalizability.

## 1. Introduction

On 11 March 2020, the World Health Organization [1] declared the novel coronavirus (COVID-19), transmitted through respiratory droplets [2], a global pandemic. By the end of March 2020, more than 100 countries (constituting more than half the worldwide population) invoked social distancing orders to limit the spread of COVID-19 [3]. The severity of preventative measures varied considerably by country (and region to region within some countries). Examples of some of the most stringent control measures include widespread closures of all non-essential businesses and schools, cancellation of public events, restrictions for in-person gatherings, closure of public transit, and stay-at-home orders with minimal exceptions (only leaving the home for essential purposes, such as groceries) [4]. As a result, individuals from across the world were forced to deal with unprecedented changes to their work, education, travel, and leisure behaviours [5,6]. Given that countries varied greatly in their initial efforts to control the spread of COVID-19, it is important to understand the impacts such efforts have had of their respective population, particularly should additional lockdowns and stay-at-home measures be invoked or, at minimum, to provide valuable information and direction in case of future pandemics [5].

Within Canada, a series of federal, provincial, territorial, and municipal preventative measures, including physical distancing [7] and widespread closures of borders, schools, and businesses [8] were implemented in mid-March (13th–20th) 2020. Additionally, stay-at-home orders (i.e., only leaving the home for essential purposes) were enacted, resulting in drastic changes to work (e.g., working from home, temporary loss of employment) and/or school (e.g., homeschooling, virtual learning) for many Canadians [8].

More than a year later (i.e., May 2021), long-term unemployment numbers remained considerably higher (+166.8%) than pre-pandemic levels (i.e., February 2020) [9]. Moreover, the Angus Reid Institute [10] suggested that almost one-third of Canadians experienced boredom as the pandemic progressed, job losses mounted, and preventive measures (e.g., stay-at-home orders, physical distancing) continued. As a result of such pandemic-related restrictions, Canadians reported spending more time at home at the onset of the pandemic, which inadvertently led to an increase in recreational screen time (i.e., screen use while sedentary that occurs during discretionary time) [7,8,9,11].

Prior to the COVID-19 pandemic, Canadians enjoyed large amounts of recreational sedentary time, with results from the 2012 Canadian Community Health Survey revealing that 57.7% of Canadian adults spent 20 plus hours per week (i.e., high risk) engaging in sedentary activities [12]. More specifically, 25.7% and 11.0% of Canadian adults spent 15 plus hours watching television (i.e., high risk) and 20 plus hours using a computer (i.e., high risk), respectively [12]. In comparison, data collected amongst a sample of 1500 Canadian adults at the onset of the COVID-19 pandemic suggests that recreational screen time increased even further as a result of the pandemic [13]. Specifically, 59% of Canadians adults in the study reported watching videos, movies, and television, and/or listening to podcasts more often at the onset of the pandemic than they did pre-COVID-19 (i.e., April 2020), whereas 41% and 24% said they were spending more time on social media and playing video games, respectively [13].

Canadian adults have cited a number of reasons for increasing recreational screen time behaviours during the pandemic. Namely, Canadians were, at least initially, invested in staying up to date with the latest pandemic-related news [9]. Additionally, physical distancing and stay-at-home orders forced Canadians to limit trips outside the home, which resulted in missed family events and in-person activities and an increase in recreational screen time opportunities [14]. Specifically, at the onset of the pandemic, Canadians spent more time on the computer, playing video games, and watching television, compared to before the pandemic [13].

Moreover, Canadian adults reported increases in social media related recreational screen time [15]. According to Watson [16], in addition to an average of 5 h and 53 min per day on the Internet and 3 h and 20 min per day watching television, Canadians spent an average of 1 h and 49 min per day on social media prior to the COVID-19 pandemic. Following the declaration of the pandemic, 41% of Canadian adults began spending more time on social media than prior to the pandemic [13], with YouTube, Instagram, LinkedIn, Twitter, and Pinterest experiencing increased daily usages [15] of 16%, 8%, 7%, 5%, and 4%, respectively [17].

Along with spending more time on social media, Canadians were also prompted to turn to alternative methods of communication to remain socially connected due to physical distancing measures [18]. As daily physical interactions with people were became limited, technology-based communication methods, such as video calls, offered great opportunities to maintain social connectedness [18]. Specifically, through the use of the video communications software (e.g., Zoom, Microsoft Teams, Google Meet), individuals were able to stay virtually social with happy hours [19,20], dinner parties [20], trivia nights [19], and birthday parties [19].

Although several sources capturing changes to Canadian’s recreational screen time behaviours at the onset of the COVID-19 pandemic exist, research extending past this initial period is limited. Therefore, the purpose of this study is to investigate how recreational screen time, and specific screen time behaviours (i.e., television time, social media, Internet use, video games, and using screens for socially connecting with others), changed throughout the first six months of the COVID-19 pandemic.

## 2. Materials and Methods

### 2.1. Procedures and Participants

#### 2.1.1. Initial Month

During the month of April 2020, adults (aged 18 and older) who regularly wear activity trackers (e.g., Apple Watch, Garmin, FitBit) were recruited to participate in a physical activity and lifestyle behaviours study via social media advertisements. Prior to data collection, written consent from the University of Windsor Research Ethics Board (REB# 20-069) was obtained. The research team shared the advertisements using their personal accounts on Instagram, Facebook, and Twitter, while encouraging their friends and followers to share the post. Those interested in participating were asked to contact the research team via email. Upon contact, prospective participants were emailed an information letter, given an identification number, a survey link, and a fillable daily step count calendar for the prior month (i.e., participants were asked to look at their activity trackers’ associated app to retroactively report the previous month’s daily step count for each day). Reminder emails were sent out after two days if prospective participants had not yet completed the survey and/or the fillable calendar. A total of 167 prospective participants contacted the research team. Upon completion of the daily step count calendar and survey, participants had the option to be entered into a draw to win a $25 grocery or gas gift card (1 in 25 odds).

#### 2.1.2. Subsequent Months

Participants who completed the initial survey and fillable step calendar were contacted via email during the first week of May 2020, informing them the study would continue for an additional five months, and they were sent new survey links and fillable step count calendars via email for each of the next five months (May to September). Participants were given the option to withdraw at any point by either contacting the research team directly or not filling out the survey and/or calendar. Participants who chose to withdraw during any given collection month were not contacted for subsequent collection months. Upon completion of each subsequent month, participants could enter into a draw for a $25 grocery or gas gift card (1 in 25 odds).

### 2.2. Measures

#### 2.2.1. Survey

Although objectively measured physical activity (i.e., step count) was collected for the larger research project, the current study only utilized the survey data. The monthly survey was administered via Qualtrics XM (Provo, UT) and took ≈ 20–25 min to complete. Informed consent was obtained at the onset of each monthly survey. Questions were generally taken or adapted from previously validated and/or national surveys and inquired about physical activity behaviours, barriers to physical activity, stress, eating behaviours, and recreational screen time behaviours, in addition to COVID-19-related questions. To allow for pre- and mid-COVID-19 pandemic comparisons across variables, participants identified the day they began physical distancing and/or self-isolating as a result of the pandemic.

Recreational screen time behaviours on school/workdays and non-school/non-work days was asked for both “before” and “since” the onset of the COVID-19 pandemic using the following question from the 2020 edition of the Canadian Community Health Survey, “On a school or workday [On a non-school or non-work day], how much of your free time do you spend watching tv or a screen on any electronic device while sitting or lying down” [21]. Responses were graded from 1 (2 h or less per day) to 5 (8 h or more per day).

Beginning in May (through September), time spent participating in five specific screen-related sedentary activities was asked using a newly developed question, “Not including work, approximately how much time (in minutes) do you spend doing the following”, with the following activities listed “watching TV”, “social media”, “browsing the internet”, “video games”, and “socially connecting via screens (e.g., emailing, texting, video chats, group chats, etc.)”.

#### 2.2.2. Data Analysis

All data were processed using IBM SPSS Statistics (version 25). Basic demographics are reported as means and standard deviations. Due to the low level of video game play among the sample (i.e., only 20–26% reported any video game play), the variable was removed from further analysis. Five separate one-way repeated measures analysis of variances (RM-ANOVAs) were conducted to determine differences in recreational screen time (pre-COVID 19, late March/April, May-September each month), watching television (once a month from May–September), using social media (once a month from May–September), using the Internet (once a month from May–September), and using screens for socially connecting with others (once a month from May–September). Assumptions (i.e., outliers, normality, and sphericity) were considered and addressed, as needed, prior to conducting each one-way RM-ANOVA. Post hoc analyses, with Bonferroni adjustments, were completed on any significant findings.

## 3. Results

**Figure 1:**
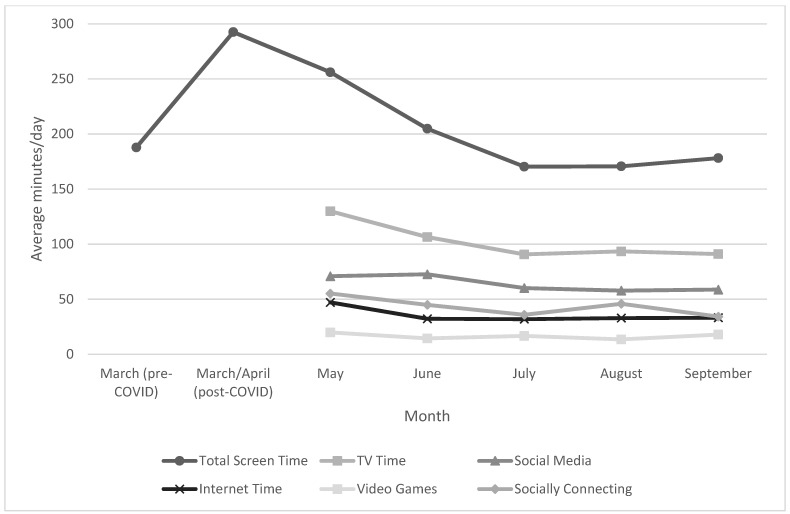
Sedentary behaviours by month.

Among the 167 people who emailed the research team (in April 2020), 142 completed the survey, with 132 of those individuals providing valid pedometer data, resulting in an initial dataset of 132 participants (107 women, 23 men, and 1 cisgender) from Canada (*n* = 11), the United Kingdom (*n* = 8), the United States (*n* = 2), and New Zealand (*n* = 1). Participants continued to submit pedometer data and complete the online survey each month: May (*n* = 95), June (*n* = 89), July (*n* = 83), August (*n* = 74), and September (*n* = 64), resulting in a final dataset of 64 participants with complete data (no pedometer data was used for the current analysis), with only Canadian participants included in the final sample. Participant demographics are available in Table 1. The majority of participants were female (87%) and Caucasian (89%), with a mean age of 39.3 ± 15.1 (range: 21 to 77 years). Figure 1 graphically displays all sedentary behaviours (i.e., total recreational screen time, watching television, using social media, using the Internet, playing video games, and using technology/screens for socially connecting with others) by month.

### 3.1. Total Recreational Screen Time

A one-way RM-ANOVA was conducted to determine differences for recreational screen time from pre-pandemic (i.e., March) to September 2020. Recreational screen time was statistically different at different time points during the first six months of the pandemic, *F* (3.882, 209.613) = 19.928, *p* < 0.001, 𝜔^2^ = 0.27. Post hoc analysis with a Bonferroni adjustment revealed that recreational screen time was statistically higher in late March/April (292.5 ± 143.0 (95% CI, 63.1 to 146.4) min/day, *p* < 0.001) and into May (256.1 ± 142.0 (95% CI, 9.6 to 126.9) min/day, *p* = 0.010), compared to pre-COVID-19 (187.8 ± 118.3 min/day).

#### 3.1.1. Watching Television

A one-way RM-ANOVA was conducted to determine differences for time spent watching television from May to September 2020. Time spent watching television was statistically different at different time points during the first six months of the pandemic, *F* (2.701, 153.932) = 6.92, *p* < 0.001, 𝜔^2^ = 0.003. Post hoc analysis with a Bonferroni adjustment revealed that time spent watching television was statistically higher in May (129.9 ± 96.6 min/day) as compared to July (90.7 ± 68.0 (95% CI, 9.9 to 68.5) min/day, *p* = 0.003), August (93.4 ± 76.7 (95% CI, 2.5 to 70.5) min/day, *p* = 0.027), and September (91.0 ± 70.0 (95% CI, 4.4 to 73.4) min/day, *p* = 0.017).

#### 3.1.2. Social Media

A one-way RM-ANOVA was conducted to determine differences for time spent on social media from May to September 2020. Time spent on social media was statistically different at different time points during the first six months of the pandemic, *F* (2.798, 151.100) = 4.678, *p* = 0.005, 𝜔^2^ = 0.050. However, post hoc analysis revealed no significant differences between timepoints.

#### 3.1.3. Internet Time

A one-way RM-ANOVA was conducted to determine differences for Internet time from May–September, 2020. Time spent on the Internet was statistically different at different time points during the first six months of the pandemic, *F* (2.236, 102.875) = 8.049, *p* < 0.001, 𝜔^2^ = 0.130. Post hoc analysis with a Bonferroni adjustment revealed that Internet time was statistically higher in May (45.87 ± 36.86 min/day) as compared to July (28.74 ± 20.30 (95% CI, 0.84 to 33.41) min/day, *p* = 0.033), August (26.53 ± 19.09 (95% CI, 5.35 to 33.33) min/day, *p* = 0.002), and September (28.11 ± 19.28 (95% CI, 2.47 to 33.7) min/day, *p* = 0.013).

#### 3.1.4. Socially Connecting

A one-way RM-ANOVA was conducted to determine differences among using screens for socially connecting with others from May–September 2020. Time spent using screens for socially connecting with others was statistically different at different time points during the first six months of the pandemic, *F* (3.083, 160.320) = 8.614, *p* < 0.001, 𝜔^2^ = 0.103. Post hoc analysis with a Bonferroni adjustment revealed that using screens for socially connecting with others was statistically higher in May (61.60 ± 41.73 min/day) as compared to July (35.79 ± 22.95 (95% CI, 8.14 to 43.48) min/day, *p* < 0.001), August (43.32 ± 29.29 (95% CI, 0.07 to 36.50) min/day, *p* = 0.048), and September (33.98 ± 26.10 (95% CI, 10.01 to 45.24) min/day, *p* < 0.001).

## 4. Discussion

To varying degrees, the COVID-19 pandemic, and its associated safety measures, resulted in changes to daily life for individuals across the globe. With considerable variability in preventative measures between countries, it is imperative to understand how country-specific measures have impacted the health of their populations. Doing so provides valuable insight into the benefits (i.e., reducing infection and death rates) and costs (i.e., to health outcomes) of each country’s strategy for virus containment, which could help inform future efforts in maintaining an optimal balance, should additional lockdowns be needed or even in the case of future pandemics.

In Canada, widespread (i.e., municipal to federal) lockdown measures to slow the spread of COVID-19 forced Canadians to spend more time at home, which resulted in changes in recreational behaviours. Specifically, among the current sample, recreational screen time behaviours increased significantly at the onset of the pandemic. However, following a slow decline over the next several months (within ≈6 months), participants appeared to return to their normal (i.e., pre-pandemic) levels of recreational screen time behaviours.

Prior to the COVID-19 pandemic, participants accumulated just over three hours/day of total recreational screen time. Conversely, in the first two months of the pandemic (i.e., late March/April and early May), total recreational screen time increased to more than four hours/day. These results are congruent with findings by Kaddatz et al. [13] and Statistics Canada [7], who also reported that Canadians increased their recreational screen time beyond pre-pandemic levels, likely as a result of government-imposed lockdown measures to limit the spread of COVID-19. Moreover, the current study’s results are congruent with Stockwell et al. [5], whose systematic review examined changes to sedentary behaviours (which includes recreational screen time) across twenty-six studies from across the world, with all reporting an initial increase in sedentary behaviour.

Specifically, increased time spent in the home may have led participants to occupy this newly increased discretionary time with recreational screen time behaviours to avoid boredom and maintain social connections with those outside the home [22]. However, unlike previous studies that only captured recreational screen time behaviours during the initial onset (i.e., first month or two) of the pandemic, the results of the current study suggest that over the course of June-September 2020, total recreational screen time among participants declined and levelled off to pre-pandemic levels. As government-imposed sanctions were loosened, it is possible that Canadians became less fearful of leaving their homes and began, once again, engaging in other non-screen related activities such as going to restaurants and using parks and trails to exercise during their recreational time [23]. Additionally, the role of pandemic “fatigue” cannot be overlooked, as many Canadians grew tired of pandemic-related restrictions, which may have resulted in the resumption of pre-pandemic behaviours, including how recreational time was spent [24].

In addition to total recreational screen time, time spent partaking in specific recreational screen-related behaviours such as watching television, using social media, going on the Internet, and engaging in virtual social connection (e.g., video-chat with family or friends) were also examined. Generally, significant initial increases in all recreational screen-related behaviours were observed at the beginning of the pandemic, with the only exception being social media use. Initial increases in specific screen-related behaviours observed in the current sample reaffirm the results of already published studies [7,8,9,13], who also reported increases in a variety of recreational screen time behaviours. However, similar to total recreational screen time, the amount of time participants spent engaging in specific recreational screen time behaviours all declined and leveled out (or close) over the course of the following six or so months to pre-pandemic levels. Again, initial increases in time spent watching television, going on the Internet, and engaging in social connection, followed by slow (return-to-normal/pre-pandemic level) declines are likely a result of the introduction of government-imposed restrictions at the pandemic’s onset limiting non-essential trips outside the home (and subsequently increasing at home discretionary time) at the onset of the pandemic follow by a loosening of such restrictions over time and the resumption of many pre-pandemic non-screen-related behaviours and activities.

Unlike other specific recreational screen time behaviours, post hoc analyses did not reveal any significant differences in social media use throughout the first six months of the pandemic, despite the production of a significant one-way repeated measures ANOVA. Given that the descriptive data suggests that participants’ social media use followed a similar trajectory to the other above-mentioned recreational screen time behaviours, the current study’s small sample and group sizes may have prevented the model from having enough power to statistically detect such differences. However, previous research by Gruzd and Mai [15], who examined the state of social media in Canada in 2020 during the beginning of the COVID-19 pandemic, reported increased social media usage amongst Canadians at the onset of the pandemic, providing support for at least the current study’s initial descriptive increase in social media usage.

### Limitations

Despite the need for research examining the longitudinal effects of the COVID-19 pandemic on recreational screen time, this study is not without its limitations. First, the study had a small sample size (*n* = 64) with the majority of participants being middle-aged Caucasian women. As a result, these results are not generalizable to the entire Canadian population. Additionally, the data for this study was collected via self-reported measures by the participants and thus is subject to recall [25] and social desirability [26] bias. Lastly, given the recruitment protocols used, the study is subject to selection bias [27], further highlighting the need to careful interpretation of study results.

## 5. Conclusions

This study is one of the first of its kind to examine Canadians’ recreational screen time behaviours longitudinally over the course of the first six months of the COVID-19 pandemic, while also contextualizing such findings with regard to pre-pandemic behaviour norms. Overall, the results suggest that despite initial increases in total and specific recreational screen time behaviours, Canadian participants showed resilience, as they were able to resume pre-pandemic levels of such behaviours once government-imposed COVID-19 restrictions began to loosen. Future studies with larger sample sizes examining recreational screen time behaviours longitudinally over the pandemic are still needed to allow for greater generalizability.

## Figures and Tables

**Table 1:** Participant demographics (*n* = 64).

Characteristic	Frequency	%
Gender		
Male	8	13
Female	56	87
Age (years)	39.27 ± 15.11 *	21–77 **
Ethnicity		
Caucasian	56	89
South Asian	1	1.5
Chinese	1	1.5
Latin America	1	1.5
West Asian	1	1.5
Other/mixed	3	5

* Mean and standard deviation; ** Range.
